# Assessing anthelmintic resistance risk in the post-genomic era: a proof-of-concept study assessing the potential for widespread benzimidazole resistant gastrointestinal nematodes in North American cattle and bison – CORRIGENDUM

**DOI:** 10.1017/S0031182020000608

**Published:** 2020-07

**Authors:** Russell W. Avramenko, Elizabeth M. Redman, John S. Gilleard

**Affiliations:** Department of Comparative Biology and Experimental Medicine, University of Calgary, Faculty of Veterinary medicine, Calgary, Alberta, Canada

The authors wish to include Dr. Claire Windeyer among the list of authors for this paper to reflect her contribution to this work. Therefore, the full list of authors should read as follows:

Russell W. Avramenko^1^, Elizabeth M. Redman^1^, Claire Windeyer^2^ and John S. Gilleard^1^

^1^Department of Comparative Biology and Experimental Medicine, University of Calgary, Faculty of Veterinary medicine, Calgary, Alberta, Canada

^2^Department of Production Animal Health, University of Calgary, Faculty of Veterinary medicine, Calgary, Alberta, Canada
